# MSProDiscuss™ Clinical Decision Support Tool for Identifying Multiple Sclerosis Progression

**DOI:** 10.3390/jcm11154401

**Published:** 2022-07-28

**Authors:** Tjalf Ziemssen, Jo Vandercappellen, Valeria Jordan Mondragon, Gavin Giovannoni

**Affiliations:** 1 Center of Clinical Neuroscience, Department of Neurology, Carl Gustav Carus University Clinic, Fetscherstraße. 74, 01307 Dresden, Germany; 2 Novartis Pharma AG, CH-4056 Basel, Switzerland; jo.vandercappellen@novartis.com (J.V.); valeria.jordan_mondragon@novartis.com (V.J.M.); 3 Blizard Institute, The Faculty of Medicine and Dentistry, Queen Mary University of London, London E1 2AT, UK; g.giovannoni@qmul.ac.uk

**Keywords:** multiple sclerosis, MS progression, clinical decision support tool, RRMS, SPMS, MSProDiscuss

## Abstract

This article describes the rationale for the development of the MSProDiscuss™ clinical decision support (CDS) tool, its development, and insights into how it can help neurologists improve care for patients with multiple sclerosis (MS). MS is a progressive disease characterized by heterogeneous symptoms and variable disease course. There is growing consensus that MS exists on a continuum, with overlap between relapsing–remitting and secondary progressive phenotypes. Evidence demonstrates that neuroaxonal loss occurs from the outset, that progression can occur independent of relapse activity, and that continuous underlying pathological processes may not be reflected by inflammatory activity indicative of the patient’s immune response. Early intervention can benefit patients, and there is a need for a tool that assists physicians in rapidly identifying subtle signs of MS progression. MSProDiscuss, developed with physicians and patients, facilitates a structured approach to patient consultations. It analyzes multidimensional data via an algorithm to estimate the likelihood of progression (the MSProDiscuss score), the contribution of various symptoms, and the impact of symptoms on daily living, enabling a more personalized approach to treatment and disease management. Data from CDS tools such as MSProDiscuss offer new insights into disease course and facilitate informed decision-making and a holistic approach to MS patient care.

## 1. Introduction

Multiple sclerosis (MS) is a chronic, potentially debilitating autoimmune-mediated neurological disorder of the central nervous system (CNS) and the most common acquired degenerative disease of the CNS in young adults [1,2]. MS pathology combines inflammation, demyelination, and axonal degeneration and results in a diverse range of functional and clinical manifestations, including physical disability, cognitive impairment, visual and sensory loss, bladder, bowel and sexual dysfunction, fatigue, and mood disorders [3,4,5]. The disease course of MS is highly variable and can be unpredictable, and individual patients may experience completely different symptoms [1,2].

In 2013, Lublin et al. updated the definitions of MS phenotypes used by the International Advisory Committee on Clinical Trials of MS as follows: “clinically isolated syndrome”, a monophasic clinical episode typical of CNS demyelination in a patient not known to have MS; “relapsing–remitting MS” (RRMS), MS with clearly defined disease relapses with full recovery or sequelae and residual deficit during recovery periods between disease relapses, characterized by lack of disease progression; “primary progressive MS” (PPMS), MS with disease progression from the onset and occasional plateaus and temporary minor improvements; and “secondary progressive MS” (SPMS), MS with an initial relapsing-remitting disease course followed by progression with/without occasional relapses, remissions, and plateaus [6]. Lublin and colleagues later clarified that “progression” refers to clinical evidence of disability worsening over time, independent of relapses, in patients who are in a progressive disease phase (i.e., with PPMS/SPMS) [7].

Patients with RRMS may not have complete recovery from clinical relapses and thus may experience an accumulation of disability over time. In up to 50% of cases, the disease gradually evolves, transitioning to SPMS over the course of 15–20 years [1,8,9]. A consensus is growing that MS exists on a continuum, with an overlap between relapsing and progressive phenotypes [5,10]. Although it is widely recognized that relapses lead to disability (relapse-associated worsening), mounting evidence demonstrates progression independent of relapse activity [11,12,13]. Progressive neuroaxonal loss is responsible for the accumulation of disability and occurs from the outset; the pathology is thought to be driven by a primary ‘smoldering’ process, accompanied by concurrent inflammatory activity reflective of the patient’s immune response to various putative causative mechanisms, including axonal and synaptic loss; demyelination; macrophage/microglial activation; oxidative injury; age-related iron accumulation; mitochondrial damage; and infection [5]. Irrespective of disease duration, the clinical presentation of MS is characterized by decreasing inflammatory activity with age (i.e., fewer relapses and new lesions on magnetic resonance imaging [MRI]); counterintuitively, the risk of developing progressive disease increases with age, implicating silent progression that does not manifest as focal lesions or relapse events [5,14,15]. Brain volume loss correlates with MS progression and is predictive of long-term cognitive decline [16,17,18]. Advanced imaging techniques can reveal useful markers of disease progression (e.g., presence of cortical lesions or active spinal cord lesions in follow-up MRI scans); however, this requires a high level of technical expertise and there is a lack of methodological standardization between different institutions [19].

At present, the Expanded Disability Status Scale (EDSS) is considered the gold standard for the assessment of neurological disability and progression in patients with MS [20]. In recent years, however, several limitations of the EDSS scale have emerged, including significant inter-rater variability and underestimation of functional parameters such as cognition, vision, and upper limb function, with changes in those parameters not always being reflected by the global EDSS score [21,22,23]. In addition, EDSS has a low sensitivity at higher values. EDSS scores >4–6 are mainly driven by changes in ambulatory function, while scores >6 are reflective of changes in general daily living [21,24]. Additional instruments have been created for more sensitive assessment of functional parameters (e.g., the Nine-Hole Peg test for manual dexterity and the Timed 25-Feet-Walk-Test for ambulation), and composite measures can be used alongside EDSS to allow a wider view of the disease [24,25]. However, such instruments can miss important signs of progression, resulting in diagnostic delays [24]. More sensitive and less subjective tools are therefore required to support the early identification of disease progression [21,26].

One of the key clinical goals of MS treatment is the prevention of the irreversible disability caused by progression to SPMS [22]. The early identification of progression is vital to optimize long-term brain health, and the ability to consistently identify progression, with a high degree of sensitivity and specificity, is directly relevant to this aim. MS management with disease-modifying therapies (DMTs) can delay the accumulation of disability [11,27,28]; however, treatment should be adapted according to the phase of the disease and, in many regions, labels of DMTs restrict use to specific phenotypic descriptors (e.g., RRMS; active SPMS) [26]. Thus, the phenotypic classification of MS, partly based on progression, has implications for treatment decisions. The time for transition from RRMS to SPMS (if it occurs) is highly variable [1,8,9], and a finite window of opportunity to identify subtle signs of progression and implement timely treatment adjustment is unique in every patient [26]. Rapid identification of MS disease progression is therefore key to facilitating treatment decisions and improving long-term prognosis [29,30]. Nonetheless, currently there is no consensus on the criteria to define disability progression, with various definitions being used in clinical trials [31]. Furthermore, few reliable clinical, radiological, or biological markers are available for early detection of the signs of progression, and the heterogeneity of the disease and lack of fully defined diagnostic and imaging criteria present additional challenges [6,20,31,32]. Certain structural biological markers, such as MRI brain volume and neurofilament light chain analysis, have been proposed to differentiate between RRMS and SPMS but no definite thresholds to separate these phenotypes have been established [20]. In particular, neurofilament light chain measurement has shown promise as a biomarker for disease activity: serum concentration correlates with new T2 and gadolinium-enhancing lesions on MRI, indicative of disease activity from active inflammation, and therefore may have some predictive value for disease progression; however, many confounding factors influence serum neurofilament light chain concentration, and further research is required to establish its prognostic value in patients with progressive and non-progressive MS phenotypes [33].

In clinical practice, patients are retrospectively diagnosed with SPMS following a period of clear progression over 6–12 months, resulting in a diagnostic delay of up to 3–4 years [6,30]. This delay can lead to brain and spinal cord damage with clear clinical consequences, including irreversible physical and mental disabilities and a significant negative impact on quality of life (QoL) [27,29]. When considering how to successfully monitor patients and identify progression, the limitations of existing tools, the lack of useful biological markers, and the heterogeneity of the signs of progression place a high level of importance on clinical evaluation, which represents a considerable challenge for neurologists [20]. An unmet need for a tool to help neurologists identify, quantify, and monitor early signs of progression in patients with MS has been widely reported [20,26,34]. 

Clinical decision support (CDS) systems, primarily used at point of care, have the potential to assist healthcare professionals (HCPs) improve medical decision-making with targeted clinical knowledge and patient and health information [35]. The development of CDS systems is a complex process, and rigorous design is required to ensure tools are clinically useful [35,36,37]. In the context of MS, the potential for CDS systems to address unmet needs around patient monitoring and identification of the early signs of disease progression is only recently being realized. CDS systems present opportunities to leverage technology to facilitate the recording and interpretation of complex data (e.g., subjective and quantitative data assessing multiple symptoms, EDSS, and MRI). 

This narrative review article covers several related topics: the articulation of an unmet need that potentially could be addressed by CDS tools designed to facilitate identification of the risk of progression in MS, description of the development of the MSProDiscuss™ (https://www.msprodiscuss.com (accessed on 19 July 2022)) CDS tool [34,38,39,40], review of the evidence in support of MSProDiscuss in clinical practice, and provision of insights into how the MSProDiscuss tool helps neurologists improve the care of patients with MS. For context, the MSProDiscuss CDS tool is a web-based application, accessible via a web browser from any device with internet connectivity, and it does not require software installation. It is designed for use by neurologists in structured consultations with patients. The tool prompts the neurologist to enter relevant demographic and clinical information and ask questions pertaining to symptoms and the impact of symptoms on daily living. It then produces an outcome report that displays scores based on each answer (allowing granular insights into symptoms and their impact on aspects of daily living) and a score synthesized by the MSProDiscuss algorithm that estimates likelihood of progression. Use of the tool in subsequent consultations allows neurologists to track progression. The development program demonstrated the MSProDiscuss algorithm has a high level of sensitivity and specificity for differentiation between RRMS and SPMS [39], and the MSProDiscuss CDS tool has been validated and tested during consultations with approximately 7000 patients with MS (n = 6974) [39,40].

## 2. The Importance and Potential Challenges of Early Identification of MS Progression

Early identification of progression in MS is key because disability progression can start early in the disease course, and delays to intervention with DMTs can have a significant impact on long-term prognosis and patient QoL [27,30]. The window of opportunity for interventional treatment to limit irreversible damage is small [41] and therefore a sensitive tool capable of detecting the early signs of disease progression is essential to maximize long-term brain health [34,38,39,40,41].

For a tool to effectively identify the progression of MS, multiple aspects of the disease should be considered, such as aspects of daily living and patient clinical history [42,43,44]. Such aspects are frequently overlooked, though research has shown that they provide neurologists with a deeper understanding of their patient’s health status [42,43]. Some composite outcome measures are effective for the detection of a broad range of clinical manifestations and can be more sensitive than measuring a single outcome such as an MRI endpoint, relapse, or disability level [24]. In concept elicitation interviews, physicians indicated a desire for a tool sensitive enough to effectively assist clinicians with the identification of MS progression [34]. Therefore, one of the challenges was to develop an algorithm able to convert complex qualitative data (from multiple domains assessed in consultation with a patient with MS, e.g., subjective symptoms, impact on QoL) into quantitative data, thereby allowing rapid, non-subjective data analysis to estimate the likelihood of progression and track longitudinal changes [38].

The MSProDiscuss tool has been developed for neurologists to use in structured consultations with their patients, to assist in monitoring the risk of progression by quantitatively assessing multidimensional data, including patient history, and to score the likelihood of progression through the use of an algorithm [34,38,39,40]. A recent study following regular use of MSProDiscuss in consultations (i.e., every 6 months) demonstrated that the tool allowed HCPs to accurately track longitudinal changes in multiple dimensions in individual patients [40].

## 3. Overview of the Development of MSProDiscuss

**Figure 1:**
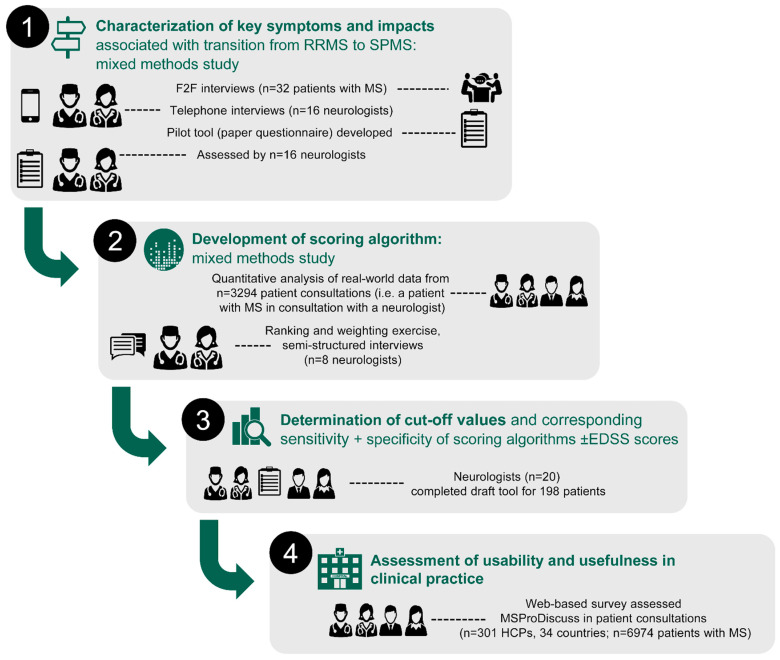
Stages in the development of the MSProDiscuss tool [34,38,39,40]. EDSS, expanded disability status scale; F2F, face-to-face; HCP, healthcare provider; RRMS, relapsing–remitting MS; SPMS, secondary progressive MS.

MSProDiscuss was developed in four stages in partnership with patients (stage 1) and HCPs (stages 1–4), as described below and summarized in Figure 1 [34,38,39,40].

### 3.1. Stage 1

The initial stage of MSProDiscuss development was to characterize the key symptoms that impact the transition from RRMS to SPMS. This was conducted using a mixed model approach, involving patient interviews and multivariate analysis of real-world data. The findings informed the selection of the key variables to be included in a questionnaire pilot tool [34].

### 3.2. Stage 2

A draft scoring algorithm was developed to determine the relevance and importance of each of the questionnaire items created in Stage 1. A novel and comprehensive approach was used to develop this draft algorithm, using data obtained from quantitative analysis of a real-world observational study, ranking and weighting exercises of variables contributing to progression, and qualitative interviews with experienced neurologists [38].

### 3.3. Stage 3

A total of 20 experienced neurologists completed a draft tool, based on interviews with 198 patients with MS (with confirmed RRMS [n = 89], with SPMS [n = 62], and suspected of transitioning to SPMS [n = 47]). These results were used to determine cut-off values and corresponding sensitivity and specificity for RRMS and SPMS identification. Excellent inter-rater reliability (intraclass correlation coefficient: 0.95 [95% CI 0.77–1.00]) and good evidence of construct validity suggested that the factors used by the draft MSProDiscuss algorithm were relevant indicators of early signs of disease progression in MS [39].

### 3.4. Stage 4

HCPs (n = 301) across 34 countries tested the MSProDiscuss tool during consultations with approximately 7000 patients with MS (n = 6974), of whom 77% (n = 5370) had RRMS. Following each consultation, the HCPs completed an initial individual questionnaire to assess the comprehensibility, usability, and usefulness of MSProDiscuss. At the end of the study, the HCPs completed a final questionnaire to capture their overall experience in using the tool, including their thoughts on its comprehensibility, usability, usefulness, and integration and adoption into clinical practice. Results from the two surveys showed that 97–98% of HCPs completed the tool within 1–4 min, and 86% were willing to integrate MSProDiscuss into their daily clinical practice. MSProDiscuss was viewed as usable and useful for facilitating clinician–patient discussions regarding MS disease progression [40].

## 4. Use and Impact of MSProDiscuss in Clinical Practice

MSProDiscuss was created to complement the work of HCPs and to facilitate the management of disease progression. The development of MSProDiscuss identified key patient data pertinent to the identification of progression. Given the heterogeneity of symptoms and experiences among different patients with MS, neurologists are sometimes required to ask a large number of questions to elucidate relevant information. MSProDiscuss supports efficient, structured consultations using a semiquantitative approach, and focuses discussions on relevant domains related to progression.

**Figure 2:**
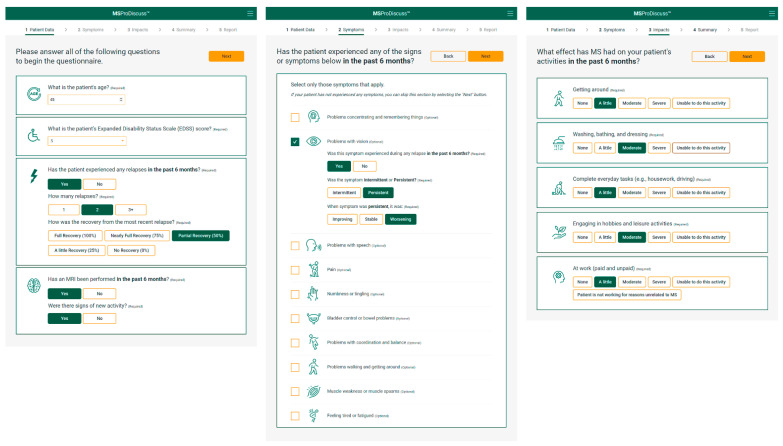
Example of the data input process performed by a physician when using the MSProDiscuss tool. Figure shows a screen shot of the tool that will become available following CE mark certification (see ‘Regulatory Classification’ section for further details) and reproduced with permission from Novartis Pharmaceuticals Corporation. Copyright 2021, Novartis Pharmaceuticals Corporation. MRI, magnetic resonance imaging.

To start, the clinician is required to input basic demographic and clinical information (current age and EDSS score, number of relapses in the last 6 months, and whether an MRI has been performed in the last 6 months). If applicable, the level of recovery from any relapses and whether there are signs of new activity on an MRI are also entered at this stage. The patient indicates which symptoms they have experienced in the last 6 months, whether they occurred during a relapse, and if they were intermittent or persistent (if persistent, then whether the symptom improved, stabilized, or worsened). Further questions then ascertain how the patient’s symptoms have affected aspects of daily living, using a scale of ‘none’ to ‘unable to do this activity’ (Figure 2).

**Figure 3:**
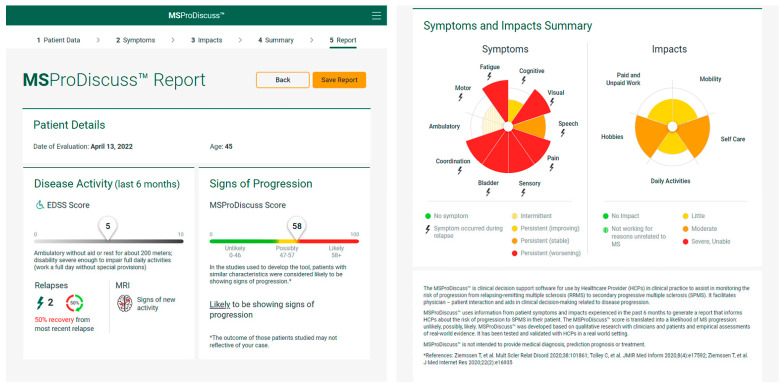
Example outcome report from MSProDiscuss. Figure shows a screen shot of the output report that will become available following CE mark certification (see ‘Regulatory classification’ section for further details) and reproduced with permission from Novartis Pharmaceuticals Corporation. Copyright 2021, Novartis Pharmaceuticals Corporation. EDSS, expanded disability status scale; MRI, magnetic resonance imaging [34,38,39].

Once completed, a score is generated from each answer and based on a weighted aggregate of these scores the MSProDiscuss algorithm synthesizes a score to estimate the likelihood of progression. An easy-to-interpret report is then generated from these results, detailing the impact the patient’s symptoms have on aspects of daily living and presenting the MSProDiscuss score for the likelihood of progression. The score can range from 0–100; scores are categorized to denote whether the patient is ‘unlikely’ (0–46), ‘possibly’ (47–57) or ‘likely’ (58–100) to be showing signs of progression (Figure 3).

Through comparison of different MSProDiscuss scores from the same patient after an interval of time (e.g., 6 months), the tool can assist in longitudinal monitoring of the risk of progression and trajectory of disease course. Thus, MSProDiscuss facilitates physician–patient interactions and aids clinical decision-making; furthermore, it can help to structure consultations to assess the impact of symptoms on daily living, thereby promoting a holistic approach to patient care, tailored to the individuals’ symptoms and experiences. Questions in the tool inquire about the impact that the patient’s symptoms have on aspects of daily living, highlighting factors known to affect QoL negatively. Consequently, neurologists may then choose to refer the patient to care services based on the individual’s needs elucidated by MSProDiscuss (e.g., physiotherapy, neuropsychology, or emotional support) for a multimodal, interdisciplinary approach. Thus, MSProDiscuss facilitates a move towards personalized medicine and a holistic approach to care. Regular use in patient consultations every 6 months allows the disease trajectory to be closely monitored and signs of progression to be identified early. In addition to early identification of progression, the capacity to track and interpret changes across multiple domains could be particularly useful in treatment decision-making. Results from the questionnaire completed by HCPs regarding usability and usefulness in clinical practice evaluations (see Figure 1, stage 4) showed that most HCPs viewed the tool as usable and useful, and agreed or strongly agreed that it would be a beneficial addition to their practice [40].

Currently, two observational, non-interventional studies (PANGAEA 2.0 evolution and AMASIA) are using the algorithm based on MSProDiscuss to monitor patients with MS [45,46]. PANGAEA 2.0 evolution also aims to gain insights into the transition between RRMS and SPMS and to identify signs of transition early in the disease course. In this study, 2000 patients with MS (with RRMS at high risk for SPMS development, n = 1000; with SPMS, n = 1000) will be observed for 2 years using the algorithm based on MSProDiscuss. To represent the standard of care, any treatment option and change of treatment are permitted, with the algorithm based on MSProDiscuss used to capture patient history [45]. Integration of the algorithm based on MSProDiscuss into future research studies, as well as into the electronic health record (EHR) and patient registries, could expedite and facilitate longitudinal collection and interpretation of multimodal patient data, ultimately improving HCPs’ ability to identify subtle signs of progression and facilitating the delivery of personalized medicine.

## 5. Regulatory Classification

In response to recent changes to medical device regulation in the European Union (EU) (Box 1), MSProDiscuss is currently being assessed as software as a medical device for CE Mark eligibility.

Box 1 **Regulatory guidelines governing clinical decision support (CDS) software in various geographical areas.**

                                                                               Europe

- The European Union (EU) recently released new medical device regulation (Regulation EU 2017/745) [47], applicable from 26 May 2021, that classifies medical devices (including standalone software as a medical device) into rule/risk-based categories, based on the consequences to the patient’s health/condition. Annex VIII, Rule 11 classifies CDS software intended to provide information of a non-life-threatening or immediate nature to support clinical decision-making as “Class IIa” (low-to-medium risk case). Class IIa products require review by a designated Notified Body for CE certification (CE Mark).
- A CE mark demonstrates that a product conforms to the general safety and performance requirements of all relevant European medical device regulations and is a legal requirement to place a device on the market in the EU [48].

                                                                          United Kingdom

- Following exit from the EU in 2020, EU medical device regulations will continue to apply until 30 June 2023 [49]. In the United Kingdom, the Medicines and Healthcare products Regulatory Agency is in the process of developing guidance for the classification of CDS software as a medical device and has taken strides to provide detailed assessment guidance for the risk-based understanding of the rules that govern CDS software [50].

                                                                          United States (US)

- In the US, medical devices are regulated using a risk-based approach through a regulatory framework governed by US Food and Drug Administration’s (FDA) Center for Devices and Radiological Health. The 2019 draft guidance on CDS software describes the FDA’s regulatory approach to CDS software functions, in line with changes suggested by the 21st Century Cures Act [51]; MSProDiscuss meets the criteria for low-risk software as defined in the guidance (criteria are based on: intended purpose, HCP as the intended user, recommendation through algorithm calculation that can be understood and independently reviewed, and determination of International Medical Device Regulators Forum (IMDRF) low-risk category I.ii.) [52]. Based on these criteria, MSProDiscuss is not subject to FDA regulation at this time.

                                                                          Other countries

- Other countries (exemplified by Canada) have regulations closely aligned with either IMDRF or EU guidelines; Brazil and Australia are currently reconsidering their regulatory framework for software as a medical device, aiming to increase regulatory scrutiny while fostering innovation.

## 6. Patient Data and Privacy Considerations for MSProDiscuss

The use of MSProDiscuss by HCPs does not require patient or HCP identifiers to be provided, recorded, or reported during discussions with the patient, and no information is stored after a session is completed. If an electronic record is desired, there is an option to download a PDF report. The use of MSProDiscuss is therefore in accordance with current EU privacy and cybersecurity requirements (i.e., the General Data Protection Regulation).

## 7. Barriers to Clinical Adoption of Digital CDS Tools

One of the main challenges that CDS tools face when introduced into a clinical environment is poor uptake and adoption. There are multiple examples of tools that were considered useful prior to roll-out but failed to be adopted in any meaningful way. There could be a variety of reasons for this, including poor usability (usually due to not involving relevant clinicians or patients in the development of the tool) and lack of appropriate dissemination [53,54]. Such potential barriers were considered throughout the development of the MSProDiscuss tool, with the long-term aim of promoting adoption.

At each stage of development, appropriate clinicians were asked to test and provide input on MSProDiscuss to create a truly useful tool (Figure 1) [34,38,39,40]. During the evaluation of usability and usefulness in clinical practice (Figure 1, stage 4), approximately 90% of HCPs thought the tool was useful for the discussion of MS symptoms and their impact on daily activities (in testing, 6121 of the 6974 (87.77%) individual questionnaires completed by HCPs after consultations showed that HCPs agreed or strongly agreed that the tool was useful; similarly, 252 of the 274 (91.9%) final questionnaires completed by participating HCPs at the end of this study also showed that they agreed/strongly agreed the tool was useful) [40].

A key barrier to the adoption of CDS tools by HCPs is the limited time allocated for consultations. Trials with MSProDiscuss indicate that the tool is nominally completed within 1–4 min (in testing, 97.3% of uses during 6974 consultations were completed within this timeframe), which was considered satisfactory [40]. Given the benefits the tool has for the care of patients with MS, there is a clear need for widespread physician education to integrate MSProDiscuss into routine clinical practice and to facilitate its long-term sustainability.

## 8. Other Tools for Assessment of MS Progression 

MSProDiscuss is not the only tool designed to support the identification of the risk of disease progression in patients with MS. The YourMSQuestionnaire (YMSQ), a 20-question patient-completed tool co-developed with patients with MS, patient advocacy groups, and clinicians has been designed to facilitate and standardize discussions between clinicians and patients [55]. The YMSQ collects patient perspectives on changes in MS symptoms, relapses, and the impact of MS on daily living activities that have occurred in the previous 6 months [56]. Patient-completed tools such as YMSQ empower patients to become involved in decision-making by ensuring that they are aware of the information that they need to participate [35]. Use of the YMSQ by patients could be paired with, and complementary to, the use of MSProDiscuss by the neurologist in consultations. The YMSQ helps patients to be well-prepared, having reflected on their symptoms, potentially resulting in faster and more comprehensive input of data into MSProDiscuss.

Another tool of interest is the SPMS Nomogram Nordics. This nomogram has been developed for research purposes to calculate the risk of a patient transitioning from RRMS to SPMS within 10, 15, and 20 years after onset of RRMS. The aim of this tool is to assist with decision-making and patient counseling in the initial phase of MS, prompting early and effective treatment for patients with a worse prognosis [57].

## 9. Future Applications of New Technologies in MS

The use of telemedicine appointments and digital tools in clinical settings has increased in recent years, mainly due to the coronavirus disease (COVID-19) pandemic [58,59]. In a recent survey of 613 patients with MS, 54% stated that they would be open to telemedicine appointments with neurologists and an unmet need for digital tools tailored to patients with MS was highlighted [60]. As MSProDiscuss is web-based, it was tested during the COVID-19 pandemic and showed promise in assisting with remote visits in which lack of face-to-face interaction can be a barrier to HCP–patient communication [40]. It is increasingly recognized that digital tools such as MSProDiscuss could facilitate a more in-depth assessment of disease evolution and progression when frequent visits to the doctor are not possible [58].

Tools developed for physicians, together with tools aimed at patients, have the potential to complement and facilitate more detailed and effective management of MS, promote shared decision-making, and empower patients by involving them in the management of their own disease [56]. Currently, telemedicine appointments remain a feasible option, especially for those who must travel to attend hospital appointments or who struggle with reduced mobility [59]. Having a digital tool that can capture the patient history and estimate the likelihood of progression remotely may improve patient outcomes by enabling appropriate assessment when face-to-face appointments are not feasible.

It has been postulated that, in the future, integration of data from multiple sources could accurately capture a patient’s characteristics, with the aim of enabling accurate modeling of disease progression and treatment simulation. This concept has been dubbed the “digital twin” [61]. Through use of an appropriate dashboard, the “twin” could facilitate discussions of pre-analyzed patient data/projections with patients, physician–patient communication, and shared decision-making [61]. The deep clinical phenotyping of patients with MS offered by the MSProDiscuss tool is a step towards the realization of the MS digital twin concept. Another way by which new technologies can be utilized, and existing technologies further leveraged, is through integration into the EHR. For example, integration of the algorithm based on MSProDiscuss could facilitate longitudinal follow-up when included as part of the clinician’s routine assessment. The use of comprehensive monitoring systems in the real world to integrate clinical, paraclinical, and patient-reported outcome data from EHRs, local databases, and patient registries could also enable a more detailed, granular description of the long-term benefits and safety of DMTs [62].

## 10. Conclusions

Identification of progression in MS can be challenging due to the heterogeneity of both the symptoms and the disease course. At present, there are no universal criteria to define disability progression in clinical trials, with a lack of clinical, radiological, and biological markers for early detection of the progressive course of the disease and poor expert consensus on specific diagnostic criteria for disability progression. Retrospective diagnosis of SPMS following 6–12 months of clear progression can delay the provision of optimum treatment for patients with active progressive disease. Widely used tools, such as EDSS, have multiple limitations, and there is a significant unmet need for a tool sensitive enough to aid clinicians with the early identification of progression effectively.

The MSProDiscuss (https://www.msprodiscuss.com (accessed on 19 July 2022)) CDS tool was developed for use by HCPs in structured consultations to assist in monitoring the risk of progression from RRMS to SPMS, thereby facilitating physician–patient interaction and aiding in clinical decision-making related to disease progression. The rigorous development of MSProDiscuss, co-created with patients and HCPs, has resulted in a demonstrably usable and useful tool. MSProDiscuss functions to support HCPs in identifying signs of early progression and, with appropriate intervention, could delay severe and irreversible disability. Integration of the algorithm based on MSProDiscuss into the EHR promises to offer insights into the disease course of MS and to facilitate informed treatment decision-making and personalized treatment.

## Data Availability

Data sharing is not applicable to this article as no datasets were generated or analyzed.
